# Bacteriocin-mediated inhibition of some common pathogens by wild and mutant *Lactobacillus* species and *in vitro* amplification of bacteriocin encoding genes

**DOI:** 10.5599/admet.1053

**Published:** 2022-02-14

**Authors:** Arooj Ahsan, Bushra Mazhar, Muhammad Kamran Khan, Madiha Mustafa, Muhammad Hammad, Nazish Mazhar Ali

**Affiliations:** 1 Department of Zoology, GCU Lahore, Pakistan; 2 Qarshi University Lahore, Pakistan; 3 University of Education, Lahore, Pakistan

**Keywords:** Bacteriocin, antibacterial activity, *Lactobacilli*, bacteriocin encoding genes

## Abstract

*Lactobacilli* are the most common probiotics used in food and other industries because of their capability of producing bacteriocins. Bacteriocins are compounds that are used to kill pathogenic microorganisms. As most bacteria have become resistant to synthetic antibacterial tools, the importance of using probiotics as antibacterial agents has increased. This work was done to check the bacteriocin effect on some common pathogens and the influence of mutation on the bacteriocin activity of *Lactobacilli* was also investigated. Four strains were isolated, identified from meat and pickles samples via culturing methods, staining, biochemical tests, and ribotyping. Preliminary tests, including Gram staining and catalase test, were done for the confirmation of *Lactobacillus* species. All strains were gram-positive and catalase-negative. Antibacterial activity was checked against *Pseudomonas aeruginosa*, *Staphylococcus aureus*, *Bacillus thuringiensis*, *Escherichia coli*, and *Salmonella enteritis* via agar well diffusion method. The mutations were done using ethidium bromide and the influence of wild and mutants were also checked. Interestingly, mutants developed more virulence than wild ones. It was also observed that they all were sensitive to pepsin. Protein estimation was done via Bradford method. Ribotyping of GCU-W-PS1 revealed 99 % homology with *Lactobacillus plantarum* and GCU-W-MS1 to *Lactobacillus curvatus* (99 % homology). Curvacin A, sakacin P, and plantaricin A genes were also amplified using specific primers. Gene sequence showed the presence of curvacin A gene in GCU-W-MS1. It was concluded that lactic acid bacteria could be used as antibacterial tools against common pathogens.

## Introduction

Bacteriocins are compounds produced by different bacterial strains and are ribosomally synthesized antimicrobial proteins. Their function is to inhibit the growth of similar or closely related bacterial strains [1]. Many lactic acid bacteria are found infrequently fermented or non-fermented foods, producing a high variety of bacteriocins. Some of the bacteriocins even have the potential to preserve food, which helps foods preserve nature and not only preserve but also restore the nutritional properties of food. In industries, we preserve food by using chemicals, though it is successful, the quality of food becomes doubtful. So for this purpose, the alternative technique that could be used is applied bacteriocin and act as bio preservatives [2]. Nisin is the only bacteriocin that is broadly used for food preservation now a day. It is the most extensively implemented bacteriocin. It is vigorous against highly pathogenic and food spoilage microorganisms, including *S. aureus* and *Listeria monocytogenes* [3]. But scientists are doing work on many bacteriocins to characterize them biochemically, chemically, and genetically so that we will be able to understand their basic mode of action. As a result of which we will revolutionize the food industry [4]. By inducing mutation in *Lactobacillus* we can improve the potency at elevated temperatures, also broadening the antibacterial spectrum [5]. Mutation is a method of changing organisms genetically. Either this mutation will be beneficial for us or may make the organism more vulnerable for mankind. Mutagenesis can be done with ethidium bromide [6] and ultraviolet (UV) light [7]. Ethidium bromide is a frame-shift mutagen genotoxic and teratogen which usually become the cause of changes like frame-shift mutation, chromosomal recombination, arrested cell division and developmental problem. However, it has been noted that such types of mutations have positive effects and usually increase the efficiency of mutants in contrast to wild ones. Mutagenic activities can be optimized by knowing the type of mutagens and dosage. Mutagens specificity effects may also help in the optimization. Mutagenesis can be improved or directed to achieve the maximum frequency of enviable mutant products [8]. The objectives of the current work were to isolate, identify, and characterize the lactobacillus species from meat and pickle samples, to check the virulent effect of wild and mutant species of *Lactobacillus* against common pathogenic bacteria, and to detect bacteriocin encoding genes in different wild and mutant species of *Lactobacilli* species.

### Importance of work

Bacteria develop resistance against many antibacterial agents currently used, so it is a dire need to develop new strategies to control these pathogenic bacteria. *Lactobacilli* are used as probiotics and are now extensively studied for their antibacterial activity against common pathogens. These bacteria produce bacteriocin, which has an impact on pathogens. We studied and detected the bacteriocin encoding genes in our native lactobacilli (wild and mutant), did ribotyping, and obtained their accession number from NCBI. This is original research work and our study with native bacterial isolates.

## Materials and methods

### Sample collection

Poultry meat samples and a variety of pickles samples (homemade and companies made) were collected in sterilized bags from different shops of Sant Nagar and Krishan Nagar, Lahore, Pakistan.

### Preparation of culture media

For the isolation of *Lactobacillus* species De Man, Rogosa and Sharpe (MRS) broth (Sigma) (1.5 g of peptone, 1.5 g of beef extract, 0.75 g of yeast extract, 3 g of glucose, 0.15 g of Tween-80, 0.3 g of ammonium citrate, 0.75 g of sodium acetate, 0.03 g of magnesium sulfate, 0.0075 g of manganese sulfate and 0.3 g of dipotassium hydrogen and 150 ml of distilled water) and MRS agar medium (Sigma)(1.5 g of peptone, 0.75 g of yeast extract, 1.5 g of beef extract, 3 g of glucose, 0.15 g of Tween-80, 0.75 g of sodium acetate, 0.3 g of ammonium citrate, 0.03 g of magnesium sulfate, 0.0075 g of manganese sulfate, 0.3 g of dipotassium hydrogen phosphate, 2.25 g of agar, and 150 ml of distilled water) were prepared with neutral pH.

### Isolation of bacteria via aseptic techniques

One gram of each sample was taken and mixed with 9 ml of buffered peptone water in the flask. The content was homogenized. The samples were serially diluted and two methods, i.e., spreading and streaking, were used to isolate bacteria on prepared MRS agar medium.

### Morphological and biochemical characterization

Pure cultures were characterized by cell morphology *via* Gram staining and biochemical characterization *via* Catalase reaction. Catalase-negative isolates were further analyzed for bacteriocin production.

### Agar-well diffusion assay

The antagonism of bacteriocin-producing strains against responsive strains (gram-positive and gram-negative bacteria) is usually determined using the agar well diffusion method [9]. Potential bacteriocin producers were grown in MRS broth at 37 °C for 24 h. The size of disposable Petri plates was 60 mm x 15 mm. Responsive strains were streaked on the agar plates, dried for a few minutes, five wells of 8 mm were made, and poured with 50 μl of an overnight culture of isolated strains. The agar plates were incubated for 30 min at 4°C to allow the diffusion of bacteriocin and incubated overnight at 37 °C. Zones of inhibitions were checked after 24 h of incubation. The diameter of inhibited zones was measured [10].

### Induction of mutagenesis

Mutagenesis with ethidium bromide was done [11]. Strains of *Lactobacilli* (i.e., GCU-W-PS1, GCU-W-PS2, GCU-W-MS1, and GCU-W-MS2) were grown up to the late logarithmic phase of growth in trypticase soy broth (TSB). The cells were yielded and washed with 0.9 % NaCl solution twice and ethidium bromide with a concentration of 0.5, 1.0, 1.5 g/l were added to 2ml of each cell suspension. The mixture was aerated on a shaking incubator at 30°C and 42°C, respectively. The treated cells were incubated in 10ml TSB washed twice and re-suspended in 0.9 % NaCl solution and after serial dilution, it was spread on MRS agar plates and incubated at 30°C and 42°C for 48 h. Then isolation of mutant was done.

### Production of bacteriocins

10 ml of MRS broth was prepared in falcon tubes for each isolate and pH was adjusted to 6.0 to 7.0. Falcon’s tubes were autoclaved at 121 °C and 15 lbs. Then 100 μl of a freshly prepared broth culture of lactic acid bacteria was inoculated with a micropipette and incubated for 24 hours at 37 °C. After incubation, falcon tubes were placed in a centrifuge (BioRad) for 30 minutes at 8000 rpm at 4 °C. With the help of filter paper, the supernatant was filtered. This filtered supernatant was crude extract.

### Protein estimation by Bradford method

Bradford method [12] was used to determine protein concentrations using a spectrophotometer (BioRad). Bovine serum albumin (BSA) was taken as standard. BSA's stock solution may be prepared if we dissolve 1mg of BSA in 1ml of distilled water. Different concentration of bovine serum albumin was prepared for the preparation of the standard curve of BSA. 100 μl of each dilution of BSA was taken in test tubes and labeled according to concentration. 100 μl of distilled water was taken in the test tube as blank. Then 5 ml of Bradford reagent was added to each test tube mixed and placed at room temperature for 15 minutes. After a few minutes, the sample color changed to blue then the absorbance of the tubes was noted at 595 nm. Graph for the standard curve was plotted, taking concentration along the x-axis and absorbance along the y-axis.

### Determination of protein concentration

For preparing the Bradford reagent, we dissolved 100 mg Coomassie Brilliant Blue G-250 in 50 ml 95 % ethanol; added 100 ml 85 % (w/v) with phosphoric acid. Then diluted it to 1 liter. When the dye had completely dissolved, it was filtered through Whatman #1 paper just before use and stored at 4 °C. To determine the protein concentration of samples, test tubes were labeled according to the sample name and 100 μl of protein sample in the form of CFS was taken while the control tube contained only a growth medium (MRS broth). 5 ml of Bradford reagent was added and mixed in each test tube and then incubated at room temperature until it turned blue. The absorbance of the tubes was taken at 595nm by spectrophotometer. The absorbance was compared with standard.

### Characterization of bacteriocin

To confirm the proteinaceous nature of bacteriocin, its sensitivity against proteolytic enzymes was tested. Proteolytic enzyme with final concentration of 2mg/ml was treated with crude bacteriocin in 0.01 M phosphate buffer at pH 7.0, 0.15 ml (150 μl), phosphate buffer (0.5M, pH 7.0), 0.15 ml (150μl) of bacteriocin and 0.15 ml (150 μl) of pepsin (0.25mg/ml) were taken in a test tube. A control was run by taking only 0.15 ml (150 μl) of bacteriocin and 0.15 ml (150 μl) of distilled water. These samples were kept in an incubator for 2 hours at 37 °C and then boiled at 100 °C for 3 to 5 minutes. No zone of inhibition indicates a positive result.

### Molecular characterization

*Lactobacillus* cells from 24 h MRS broth cultures were centrifuged at 6000 rpm for 5 min to pellet out cells for DNA isolation using phenol: chloroform extraction method. Selected bacteriocin-producing strains were finally identified along the partial length of the 16S rRNA gene sequence. Amplification with 16S rRNA gene is used to identify the microbes to specie level. 50 μl reaction mixtures were used for PCR amplification using a thermocycler (BioRad1000). The polymerase chain reaction was performed for 35 reaction cycles in a thermocycler. All amplification reactions were performed in a PCR cycling system using the conditions given below: the first cycle was led by initial denaturation at 94 °C for 5 min, followed by 35 cycles, denaturation at 94 °C for 0.50 sec, annealing gradient ranging from 49.3 °C to 60.2 °C for 0.30 sec and polymerization at 72 °C for 50 sec. The reactions were terminated with 10 min of elongation at 72 °C and then chilled to 4 °C.

### Sequencing and sequence analysis

PCR product was run on 1 % agarose gel. Bands in the gel were observed in UV (BioRad). The products of PCR were sent to 1^st^ Base Laboratory, Malaysia, for sequencing. The sequences acquired were sent to the National Centre for Biotechnology Information (NCBI) to analyze the nucleotide-nucleotide BLAST data.

### Characterization of the bacteriocin structural gene

Various structurally different bacteriocins have been described to date. We performed PCR amplification experiments to determine whether the selected strains carry structural genes of known species that were the same as those of other species. In this experiment, we used specific primers for the detection and identification of specific bacteriocin genes. For PCR amplification of the specific gene, specific primers were designed based on gene sequences available in the GenBank database.

### Amplification of bacteriocin encoding genes

By performing the polymerase chain reaction, bacteriocin encoding genes of the chosen isolates were identified using different specific primer pairs, the primers for *sakacin P*, and bacterial genomic DNA. After the PCR, amplified products were analyzed by electrophoresis on 1 % agarose gel (1X TAE buffer pH 8.3) using a 100 kb DNA ladder (Enzynomics) as a molecular weight standard weight. Electrophoresis was done at 80 V for 60 min.

### Analysis of bacteriocin gene sequence

The products of PCR were sent for sequencing to 1^st^ Base laboratory, Malaysia. Nucleotide sequencing of the PCR amplicon was compared with the GenBank database. Sequences were blasted against the NCBI GenBank database. Sequence alignment was performed. The gene sequences related to bacteriocin production were deposited in the NCBI, GenBank.

## Results

### Isolation of lactic acid bacteria

Fourteen bacterial strains were isolated, eight strains of *Lactobacillus* were isolated from four samples of minced meat (P1, P2, P3, P4, P5, P6, **GCU-W-MS1**, **GCU-W-MS2)**, and six strains of *Lactobacillus* isolated from different sources of pickles (H1, **GCU-W-PS1**, H3, N1, **GCU-W-PS2**, N3) (Table 1).

### Morphological and biochemical characteristics

**Figure 1.**
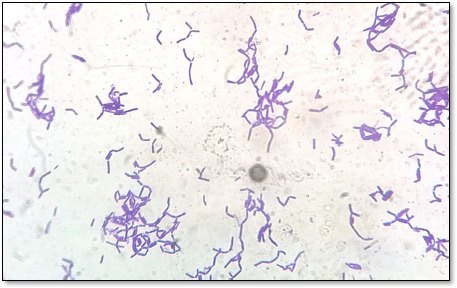
Gram staining test showing results of selected strains as a gram-positive

**Figure 2.**
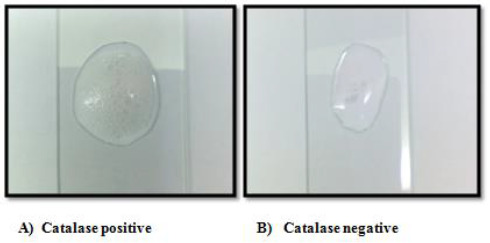
Catalase test showing negative results for selected strains

Grams staining result showed that all the isolated strains were purple-stained and were gram-positive and were rod or cocci in shape (Table 2 and Figure 1). The results revealed that all isolated bacterial strains could not degrade hydrogen peroxide and were catalase-negative (Figure 2).

### Agar well diffusion assay (inhibitory assay)

The agar well diffusion method revealed that GCU-W-MS1, MS2, GCU-W-PS1, and GCU-W-PS2 were found an active producer of bacteriocins (Table 3). **GCU-W-MS1** showed maximum inhibition of *Escherichia coli* (9 mm). On the other hand, the least or even minimum inhibition of *Salmonella enteritidis*, *S. aureus, P. aeruginosa*, and *B. thuringiensis* was recorded.

**GCU-W-MS2** showed maximum inhibition of *S. aureus* and *Salmonella enteritidis* with 8 mm zone of inhibition, while *Escherichia coli, P. aeruginosa, B. thuringiensis* were resistant to *L. johnsonii.* **GCU-W-PS1** showed maximum inhibition of *S. aureus* and *Salmonella enteritidis* with 7 mm zone of inhibition, while resistance to *P. aeruginosa*, *Escherichia coli* and *B. thuringiensis* showed less degree of inhibition with a zone of 3 mm. **GCU-W-PS2** showed a high degree of inhibition of *S. aureus* (8 mm). *P. aeruginosa* showed the least bacteriocin activity.

### Mutagenesis in Lactobacillus spp.

GCU-W-PS1, GCU-W-PS2, GCU-W-MS1 and GCU-W-MS2 strains were the species that showed maximum bacteriocin activities against pathogenic bacteria i.e., *S. aureus*, *P. aeruginosa, B. thuringiensis, Escherichia coli*, and *Salmonella enteritis.* So, the mutagenesis induction technique was being used on these strains to check either mutation makes them more reactive against a pathogen or not. Ethidium bromide is a teratogen chemical and can change the genetic makeup was being used for inducing mutation. Interestingly, it was observed morphologically and biochemically (bacteriocin assay) that mutation enhances the activity of *L. curvatus, L. plantarum*, and *Lactobacillus sakei.*

### Morphological characteristics of mutants’ v/s wild

**Figure 3.**
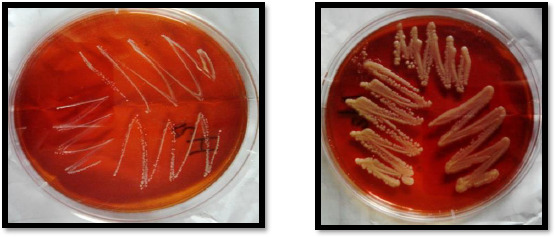
*L. curvatus* (wild and mutant)

It can be seen that after mutation, the growth of different *Lactobacillus* strains has been increased at a high rate. The increased growth rate in mutants is shown in Figure 3, which is a comparison between wild and mutant *Lactobacillus*.

### Bacteriocin assay

**Figure 4.**
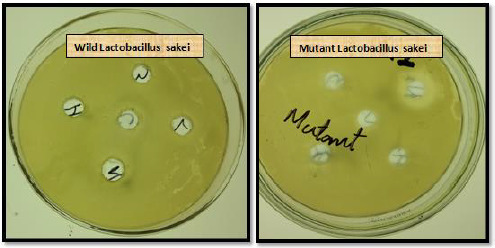
Comparison of antibacterial activity between wild and mutant species on pathogen *S. aureus*

Bacteriocin assay (agar well diffusion assay) was done again on mutant species of *Lactobacillus i.e. L. curvatus, L. plantarum, Lactobacillus sakei*, and *Lactobacillus johnsonii* (Figure 4). They showed more inhibition zone compared to wild species of *Lactobacillus* (Table 4).

### Characterization of bacteriocin

Isolated strains CPS were being treated with pepsin which is a proteolytic enzyme. When pepsin was being added, it was seen that all isolated strains showed sensitivity against it (Table 5).

### Protein estimation by Bradford method

**Figure 5.**
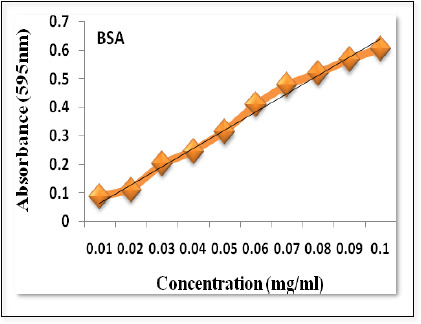
Standard curve of BSA

The standard curve of BSA was plotted (Figure 5) and protein concentration produced by Lactobacillus spp. was calculated.

### Molecular characterizations

**Figure 6.**
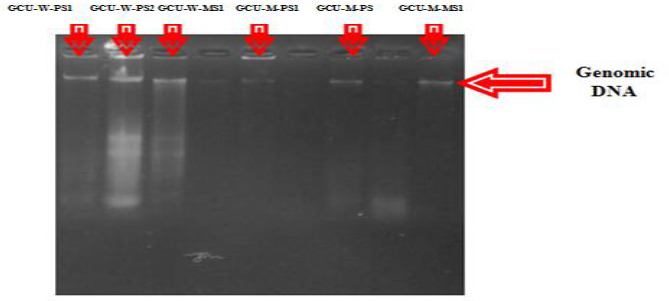
1 % agarose gel GCU-W-PS1, GCU-W-PS2, GCU-W-MS1, GCU-M-PS1, GCU-M-PS, GCU-M-MS1 indicates the genomic DNA isolated from poultry and pickle samples and their mutants

**Figure 7.**
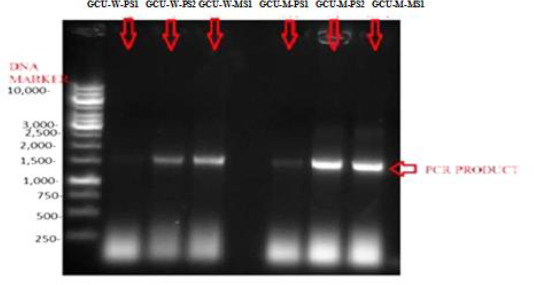
Agarose gel 1% GCU-W-PS1, GCU-W-PS2, GCU-W-MS1, GCU-M-PS1, GCU-M-PS2, GCU-M-MS1 indicates the PCR products of genomic DNA of isolated wild *Lactobacilli* from poultry and pickle samples and their mutants.

Strains with the best inhibitory activity were selected for molecular characterization (Table 6). 1 % gel was prepared and about 4μl DNA sample was loaded for gel electrophoresis sharp bands of genomic DNA of GCU-W-PS1, GCU-M-PS1, GCU-W-PS2, GCU-M-PS2, GCU-W-MS1 and GCU-M-MS1 were visualized (Figure 6). Universal forward and reverse primers were used to amplify genomic DNA and 1 % agarose gel was being used to check PCR product (Figure 7).

### Sequencing and sequence analysis

For the sequencing of PCR products, the samples were sent to 1^st^ Base Laboratory, Malaysia. It was found that GCU-W-PS1 (wild) and GCU-M-PS1 (mutant) have 97 % and 99 % resemblance with *L. plantarum* respectively. GCU-W-MS1 (wild) and GCU-M-MS1 (mutant) strains showed a resemblance 98 % with *L. curvatus* (Table 6).

### Characterization of bacteriocin encoding genes

**Figure 8.**
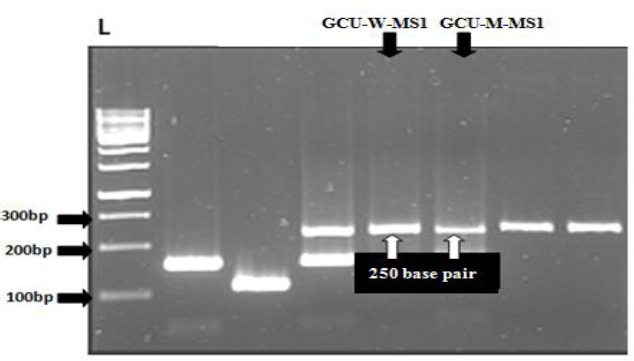
Agarose gel electrophoresis of PCR fragments generated with *curvacin A* specific primer from DNA of selected GCU-W-MS1 and GCU-W-MS1m strain (*L. curvatus)*

Using specific primers designed for curvacin, sakacin, and plantaricin genes, strains that showed maximum antibacterial activity were amplified. PCR results had shown GCU-W-MS1 (wild) and GCU-M-MS1 (mutant) (*L. curvatus*) strain amplified for curvacin gene and showed 100 % homology (Figure 8).

## Discussion

Food safety is becoming a big challenge now a day. In the food industry, the use of chemical preservatives has a positive effect; however, they have some negative impacts on the health of human beings [13]. Modern society is more concerned about food safety as chemicals and artificial additives are eliciting poisonous concerns. So era demands to use natural sources for bio-safety and health. Bacteriocins are the natural compounds found in probiotics, especially LAB, i.e., mostly *Lactobacillus* species [14]. These compounds can kill pathogens found in food, preserve it, and avoid the degradation of food. And they were generally recognized as safe (GRAS). Usually, *Lactobacillus* bacteria can be isolated from yogurt, pickles, and meat. Bacteriocin produced by *Lactobacillus* promises safe to use as a food preservative found in vegetables, cheese, dairy products, and meat, as they inhibit pathogenic contamination during the processing [15]. They show high antimicrobial activity against disease-causing bacteria [16]. The inhibition zone can be counted as positive if the zone is 2 mm or greater than 2mm. And the present study shows that the strains i.e. GCU-W-PS1, GCU-W-PS2, GCU-W-MS1, and GCU-W-MS2, exhibit a greater inhibition zone of more than 2 mm. The highest degree of inhibition that was measured is more than 9 mm. GCU-W-MS2 was the strain isolated from meat that had shown a high degree of inhibition on the culture plate of *S. aureus* 9.2 mm. However, a strain isolated from pickles named GCU-W-PS1 showed resistance for *P. aeruginosa.* [17] used well diffusion method to ensure the antibacterial activity of lactic acid bacteria of raw milk of cattle against *S. aureus, Bacillus mycoides, Proteus valgaris*, and *Klebsiella pneumonia*, these pathogens were found to be sensitive for bacteriocin produced by *Lactobacillus* species [18]. As these strains showed more bacteriocin active results, they were selected for the induction of mutagenesis to check whether a mutation will increase the efficiency against a pathogen. The results have shown that mutagenesis increases the efficiency in some bacteria i.e., GCU-W-PS1 which had no bacterial activity and exhibited no inhibition zone. However, present work showed that after mutation, the pathogenicity of GCU-M-PS1 increased, showing an inhibition zone of 4mm against *P. aeruginosa.* By site-directed mutagenesis in bacterial structural genes and with the help of genomics and proteomics, this could be made possible to construct a new family with peptides to increase the antibacterial activity [19]. Biochemical testing (i.e., sensitivity to proteolytic enzymes and protein estimation by Bradford method) and molecular characterization have been proved that mutant strains of *Lactobacillus* have more virulence against pathogens. In [20], a large number of Gram-positive bacteria, including food-borne pathogen *Listeria monocytogenes, Bacillus cereus*, and *S. aureus*, were inhibited by bacteriocin produced by *L. plantarum.* The analysis of LAB bacteria from poultry meat and pickle in this study indicated several bacterial genera with the ability of bacteriocin production [21]. In conclusion, the P11 strain (*L. curvatus*) has bacteriocin encoding gene exhibited inhibitory activity against pathogenic bacteria. Time-saving methods must be used for characterization. One of them is PCR which is used for rapid identification. Through the PCR bacteriocin encoding, genes were detected. The strains P7, P11 were amplified with already reported primers. Generally, one species of bacteria may produce more than one type of bacteriocin and one bacteriocin may not produce by single species of bacteria. Two subspecies of *L. plantarum* isolated from different sources, such as fermented sausages and cucumber fermentation were shown to have the same plantaricin encoding gene PlnA [22,23]. *Curvacin A* encoding gene was identified in both the *L. curvatus* and *Lactobacillus sakeii* [24]. In the present study, the GCU-W-PS1 (*L. curvatus*) strain was amplified with *curvacin A* gene.

## Conclusion

The ability to produce bacteriocins provides the antibacterial potential to lactic acid bacteria against various common pathogens like *Pseudomonas aeruginosa, Staphylococcus aureus, Bacillus thuringiensis, Escherichia coli*, and *Salmonella enteritis*. They show antibacterial activity against both their wild and mutant strains. Bacteriocins are safe to use with a minimum negative impact on human health because they are natural products and should be considered safe for food and preservation in the food and dairy industries.

## Figures and Tables

**Table 1.** Isolated *Lactobacilli* strains from poultry meat and pickle sample

Nature of sample	Isolated strains
Minced poultry meat (Sant nagar)	P1, P2
Minced poultry meat (Sant nagar)	P3, P4
Minced poultry meat (Krishan nagar)	P5, **GCU-W-MS1**,
Minced poultry meat (Krishan nagar)	P6, **GCU-W_MS2**
Pickle (mixed) Home made	H1,**GCU-W-PS1**
Pickle (mixed) Shan company	H3,
Pickle (mixed) Shangrilla company	**GCU-W-PS2**
Pickle (mixed) Shezan company	**GCU-W-PS2**, N3

**Table 2.** Morphological and biochemical characterization of bacterial isolates

Strains	Colony color	Colony size	Colony texture	Gram Staining	Catalase test
P1	White	medium	Wet	+ive	-ive
P2	White	medium	Wet	+ive	-ive
P3	Offwhite	small	Wet	+ive	-ive
P4	Offwhite	medium	Wet	+ive	-ive
P5	White	small	Wet	+ive	-ive
P6	White	small	Wet	+ive	-ive
GCU-W-MS1	Offwhite	medium	Dry	+ive	-ive
GCU-W-MS2	Offwhite	small	Wet	+ive	-ive
H1	White	small	Wet	+ive	-ive
GCU-W-PS1	White	small	Wet	+ive	-ive
H3	Offwhite	medium	Wet	+ive	-ive
N1	Offwhite	medium	Wet	+ive	-ive
GCU-W-PS2	Offwhite	medium	Wet	+ive	-ive
N3	White	small	Wet	+ive	-ive

**Table 3.** Inhibitory activity of bacteriocin of wild strains of *Lactobacillus* isolated from poultry meat and pickles against some pathogenic bacteria

Sample no.	Zone of inhibition (mm)				
	*Salmonella enteritidis*	*P. aeruginosa*	*Escherichia coli*	*S. aureus*	*B. thuringiensis*
	M ± S.E				
GCU-W-PS2	5.1 ± 00.10	2.0 ± 00.10	3.0 ± 00.10	7.9 ± 00.10	2.0 ± 00.20
GCU-W-PS1	6.0 ± 00.10	Resistant	2.7 ± 00.10	7.5 ± 00.20	3.5 ± 00.10
GCU-W-MS1	8.2 ± 00.20	2.1 ± 00.13	8 ± 00.15	8.0 ± 00.10	2.7 ± 00.13
GCU-W-MS2	7.8 ± 00.10	3.3 ± 00.10	5.4 ± 00.10	9.2 ± 00.14	2.4 ± 00.10

**Table 4.** Inhibitory activity of bacteriocin of mutant strains of *Lactobacillus* isolated from poultry meat and pickles against some pathogenic bacteria

Sample no.	Zone of inhibition (mm)				
	*Salmonella enteritidis*	*P. aeruginosa*	*Escherichia coli*	*S. aureus*	*B. thuringiensis*
	M ± S.E				
GCU-W-PS2	5.5 ± 00.16	2.0 ± 00.10	4.0 ± 00.10	7.0 ± 00.10	2.0 ± 00.20
GCU-W-PS1	9.0 ± 00.18	4.0 ± 00.10	5.0 ± 00.10	7.0 ± 00.30	3.0 ± 00.10
GCU-W-MS1	7.0 ± 00.25	2.1 ± 00.39	5.0 ± 00.15	7.8 ± 00.14	3.0 ± 00.13
GCU-W-MS2	8.0 ± 00.10	Resistant	6.0 ± 00.10	9.6 ± 00.18	2.1 ± 00.10

**Table 5.** Sensitivity of bacteriocin of bacterial isolates isolated from poultry meat and pickle towards pepsin

Serial no.	Isolated bacterial strains	Sensitivity to Pepsin (proteolytic enzyme)
1.	GCU-W-PS1 (*L. plantarum*)	Sensitive
2.	GCU-W-PS2 (*Lactobacillus sakei)*	Sensitive
3.	GCU-W-MS2 (*Lactobacillus johnsonii)*	Sensitive
4.	GCU-W-MS1 (*L. curvatus)*	Sensitive
5.	GCU-M-PS1 (*L. plantarum*)	Sensitive
6.	GCU-M-PS2 (Lactobacillus sakei)	Sensitive
7.	GCU-M-MS2 (*Lactobacillus johnsonii)*	Sensitive
8.	GCU-M-MS1 (*L. curvatus)*	Sensitive

**Table 6.** Accession number given to identified strains

Sr. no.	Strain name	Identified species name	Accession no.
01	GCU-W-PS1	*Lactobacillus plantarum*	KY048156
02	GCU-M-PS1	*Lactobacillus plantarum*	KY048157
03	GCU-W-MS1	*Lactobacillus curvatus*	KY048158
04	GCU-M-MS1	*Lactobacillus curvatus*	KY048159
